# MIDER: Network Inference with Mutual Information Distance and Entropy Reduction

**DOI:** 10.1371/journal.pone.0096732

**Published:** 2014-05-07

**Authors:** Alejandro F. Villaverde, John Ross, Federico Morán, Julio R. Banga

**Affiliations:** 1 Bioprocess Engineering Group, IIM-CSIC, Vigo, Spain; 2 Department of Chemistry, Stanford University, Stanford, California, United States of America; 3 Department of Biochemistry and Molecular Biology, Complutense University, Madrid, Spain; University of Manchester, United Kingdom

## Abstract

The prediction of links among variables from a given dataset is a task referred to as network inference or reverse engineering. It is an open problem in bioinformatics and systems biology, as well as in other areas of science. Information theory, which uses concepts such as mutual information, provides a rigorous framework for addressing it. While a number of information-theoretic methods are already available, most of them focus on a particular type of problem, introducing assumptions that limit their generality. Furthermore, many of these methods lack a publicly available implementation. Here we present MIDER, a method for inferring network structures with information theoretic concepts. It consists of two steps: first, it provides a representation of the network in which the distance among nodes indicates their statistical closeness. Second, it refines the prediction of the existing links to distinguish between direct and indirect interactions and to assign directionality. The method accepts as input time-series data related to some quantitative features of the network nodes (such as e.g. concentrations, if the nodes are chemical species). It takes into account time delays between variables, and allows choosing among several definitions and normalizations of mutual information. It is general purpose: it may be applied to any type of network, cellular or otherwise. A Matlab implementation including source code and data is freely available (http://www.iim.csic.es/~gingproc/mider.html). The performance of MIDER has been evaluated on seven different benchmark problems that cover the main types of cellular networks, including metabolic, gene regulatory, and signaling. Comparisons with state of the art information–theoretic methods have demonstrated the competitive performance of MIDER, as well as its versatility. Its use does not demand any a priori knowledge from the user; the default settings and the adaptive nature of the method provide good results for a wide range of problems without requiring tuning.

## Introduction

Reverse engineering a network consists of inferring the structure of interactions between its components from a set of data. This problem appears in many different contexts, such as chemistry (construction of chemical reaction mechanisms), biology (inferring gene regulatory networks), engineering (system identification), or social sciences [1]. In bioinformatics, the network inference problem consists of reconstructing the structure of a cellular network from data. Cellular networks can be classified as gene regulatory, metabolic, or protein signaling, depending on the type of entities and interactions. Methods developed specifically for a particular type of network usually try to exploit previously available knowledge, and make assumptions about the underlying structure; a typical example is inference of gene regulatory networks (GRN) [2]–[11]. However, there is also a number of methods that are not tailored to a particular type of network, and are applicable to chemical reaction networks of any kind [12], [13].

Reviews of network inference methods typically find large discrepancies among the predictions of different algorithms, and usually conclude that there is no single best method for all problems [5], [14]. Different methods highlight different interaction types and can be therefore considered complementary [15]–[17]. Furthermore, even the best methods achieve low prediction accuracies, and manage to recover only small networks of simple topology [10]. Hence it has been argued that accurate reconstruction of large-scale regulatory network from expression data alone is currently not feasible, and unsupervised inference methods should focus instead on smaller-scale networks for which higher-quality data is available [10].

The present work addresses the problem of recovering the structure of a network from the available data in its most general form. This entails that no assumptions about the underlying structure are made, and previous knowledge is not taken into account. Interactions should be deduced only from the statistical features of the data, without resorting to biological intuition. To reach this goal, many methods have exploited the analytical tools provided by information theory. The fundamental concept of information theory is entropy, which was introduced by Shannon [18] as a way of measuring the uncertainty of a random variable. Let X be a discrete random vector with alphabet 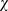 and probability mass function 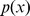. The entropy is
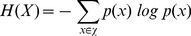
(1)where log is usually the logarithm to the base 2. In the case of continuous variables the 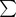 are replaced by 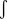. The joint entropy of a pair of variables (X,Y) is 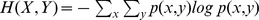. Conditional entropy 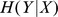 is the entropy of a random variable conditional on the knowledge of another one:



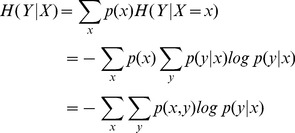
(2)The joint entropy and the conditional entropy are related so that 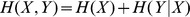.

The relative entropy, which is also known as Kullback–Leibler divergence or information gain, is a measure of the distance between two distributions. It is defined as 
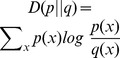
; it is always non-negative, and it is zero if and only if 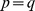. The relative entropy between the joint distribution, 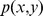, and the product distribution, 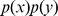, is called mutual information, 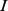 [19], that is,
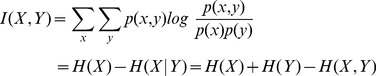
(3)


The mutual information measures the amount of information that one random variable contains about another. In other words, it is the reduction in the uncertainty of one variable due to the knowledge of another. Since it does not assume any property of the dependence between variables–such as linearity or continuity–it is more general than linear measures such as the correlation coefficient, and is able to detect more interactions [20]. The concept of mutual information suggests its application for inferring interaction networks of any kind (chemical, biological, social): if two components of a network interact closely, their mutual information will be large; if they are not related, their mutual information will be theoretically zero.

In the next section (Methods) we present a methodology and software toolbox called MIDER (Mutual Information Distance and Entropy Reduction). MIDER seeks to achieve high precision on small and medium-scale networks of any kind, cellular or otherwise, although it can also be applied to large-scale problems. It is designed with the aim of accurately distinguishing between direct and indirect interactions, thus minimizing the number of false positives. In the Results section the performance of MIDER is compared with that of four other methods reviewed in this Introduction, using seven benchmark problems. Final remarks are given in the Conclusions section.

## Methods

**Figure 1:**
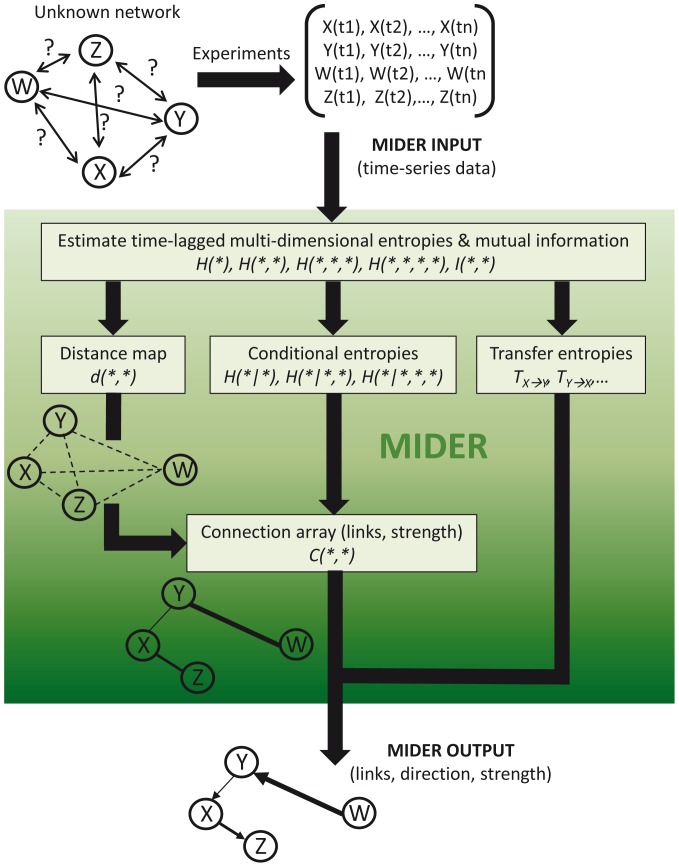
MIDER workflow.

The MIDER workflow is shown in Figure 1. It begins by estimating time-lagged multi-dimensional entropies and mutual information from data. These estimates are then used for constructing a distance matrix between variables, based on estimates of the mutual information from data. This matrix is converted for visualization into a two-dimensional map of the variables (species), with the distances among them being a first guess for their connections (reactions, interactions). Then an entropy reduction step based on conditional entropies is applied to further refine the map, helping in discriminating between direct and indirect connections. Finally, the direction of the inferred links is assigned using transfer entropies. The next subsection (Background) gives an overview of the information-theoretic methods already available, and the subsequent subsections present the details of the MIDER methodology.

### Background: information-theoretic methods for network inference

A recent review on information-theoretic network inference methods can be found in [21]. Early examples of biological applications, which relied basically on the definition of mutual information, Equation (3), can be found in [22]–[26]. More refined techniques appeared soon afterwards, such as the Entropy Metric Construction (EMC) presented in [27], [28], which is oriented towards reverse engineering chemical reaction mechanisms. It estimates mutual information from time series data of concentrations of the species, and defines the distance between two species X and Y as 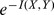. Since it takes into account possible time delays (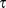) between species, the EMC distance is actually the minimum regardless of 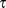:
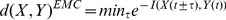
(4)


Thus it defines a matrix of distances between species, and by applying Multidimensional Scaling (MDS) it obtains a two-dimensional map that serves as an indication of species connectivity. EMC was designed as a generalization of a previous method called CMC, Correlation Metric Construction [29], [30], which used correlations instead of mutual information and extracted indications of the causality of reactions from the temporal ordering of the correlation maxima. This network inference approach based on time–lagged correlations was combined in [31] with an additional parameter estimation step, where the kinetic rate constants resulting from the guessed interactions were also deduced. Samoilov et al proposed to extend EMC with the Entropy Reduction Technique, ERT [27], [28]. This never tested method was designed to return the ordered list of species X* with which a given species Y reacts, exploiting the property that, if a variable Y is completely independent of a set of variables **X**, then theoretically 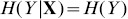; otherwise 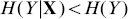. The ERT algorithm starts with an empty set of reacting species, 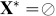, for every species Y. Then it finds the species that causes the largest entropy reduction, 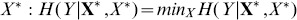, and adds it to the set, 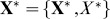. This is repeated until 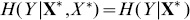, or when all species except Y are already in X*. In other words, ERT determines whether the nonlinear variation in a variable Y is explainable by the variations of a subset of the other variables in the system, X*. This is carried out by iterating through cycles of adding a variable X* to X* that minimizes 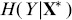 until further additions do not decrease the entropy.

A different way of distinguishing direct from indirect interactions is carried out by the ARACNE method [32], [33], which builds on [26] and includes an additional step. It was designed for identifying transcriptional interactions between gene products, using microarray expression profile data. It applies the Data Processing Inequality (DPI) to discard indirect interactions. The DPI is a property of mutual information [19] that states that, if 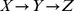 forms a Markov chain, then 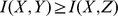. The ARACNE algorithm examines each gene triplet for which all three mutual informations are greater than a threshold 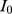 and removes the edge with the smallest value. An extension called hARACNe, which considers indirect interactions of higher-order (that is, mediated by more than one extra regulator), has recently been published [34]. Additionally, a time-delay version of ARACNE, TD-ARACNE [35], can be used when time-series data is available.

Context Likelihood of Relatedness, CLR [20], is another technique designed for inferring transcriptional interactions. It estimates the mutual information between a transcription factor 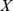 and a gene 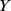, and corrects its value by comparing it with the background distribution of mutual information for all possible interactions involving 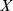 or 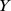. CLR takes into account the network context, assuming that the most probable interactions are not those with the highest MI scores, but those whose scores are significantly above the background distribution. The main purpose of this correction step is to remove false correlations. CLR was tested using *E. coli* data and known regulatory interactions from RegulonDB; for that data set it was reported [20] that it outperformed other methods, including ARACNE.

The Minimum Redundancy Maximum Relevance method (MRMR) introduced in [36] combines two criteria. On the one hand, it aims at selecting the subset of genes that have the maximum relevance for a given target, while on the other hand it aims at selecting genes that are mutually maximally dissimilar (minimum redundancy). MRNET [37] is a method for inferring transcriptional networks that applies the MRMR idea. It seeks to maximize, for every target variable Y, a score 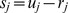 which consists of a relevance term 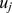 and a redundancy term 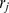, which are defined as
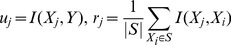
(5)


The rationale is to rank direct interactions better than indirect interactions. MRNET was implemented in the R package MINET [38], which also includes implementations of ARACNE and CLR.

Another way of discriminating between direct and indirect interactions is given by MI3, three-way mutual information [39]. It is a statistical learning strategy specifically designed to detect cooperative activity between two regulators in transcriptional regulatory networks. It aims at detecting higher order interactions, a purpose for which it uses scores calculated from multiple-variable joint entropies. Given three variables 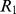, 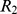, and 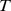, where 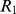 and 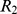 are possible regulators of the target variable 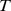, the MI3 metric is defined as
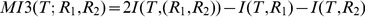
(6)


Finally, some authors have proposed to redefine the concept of entropy in order to make it more suited for inferring networks where long-range interactions exist. Equation (1) is the classical definition of entropy, also known as Boltzmann-Gibbs entropy (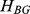) or Shannon entropy. This concept is the basis of standard statistical mechanics, which applies to physical systems that satisfy ergodicity at the microscopic dynamical level. Standard statistical mechanics is extensive: it assumes that, for a system 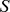 consisting of 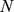 independent subsystems 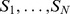, it holds that 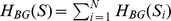. Tsallis [40] argued that systems with long-range interactions violate this hypothesis, and proposed to overcome this limitation by generalizing 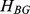 as:
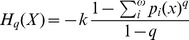
(7)where 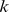 is a positive constant that sets the dimension and scale, 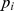 are the probabilities associated with the 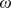 distinct configurations of the system, and 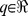 is the entropic parameter. The entropic parameter characterizes the degree of nonextensivity, which in the limit 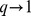 recovers 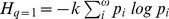, with 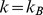, the Boltzmann constant. The generalized entropy 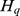 is non-extensive for systems without correlations; however, for complex systems with long-range correlations the reverse is true: 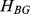 is non-extensive and 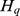 becomes extensive [41]. By defining the 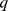-logarithm function as 
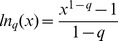
, the generalized entropy can be expressed in a similar form as the Boltzmann-Gibbs entropy of Equation (1), 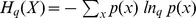, and, as in Equation (3), a generalized mutual information can be defined [42],
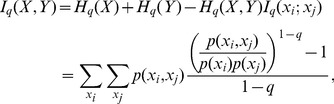
(8)which has the necessary properties to be used as a criterion measure for consistent testing [43]. The generalized conditional entropy is 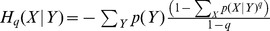. It is possible to look for dependencies between 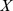 and 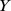 by minimizing 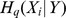, as done by Lopes et al [44] in the context of reverse-engineering gene networks. They reported an improvement on the inference accuracy by the adoption of subextensive entropies, which reduced the number of false connections.

### Calculating mutual information

This subsection explains how MIDER (1) estimates mutual information from data using an adaptive partitioning algorithm, (2) provides several normalizations of the mutual information, and (3) plots three-dimensional landscapes of the mutual information pairs as a function of the time lag between variables.

#### Estimation of mutual information from data

Mutual information can be either analytically calculated or estimated from experimental data. For reverse engineering purposes, knowledge of the underlying system equations cannot be assumed; therefore it is necessary to estimate mutual information from the available datasets. This is far from trivial, and several algorithms have been proposed for this task. The simplest one is a naive estimation, where the data is binned into equally sized intervals and an indicator function 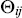 counts the number of datapoints within each bin. Then the probabilities are estimated from the relative frequencies of occurrence,
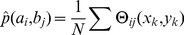
(9)


This simple approach gives good results if the number of data points is large; otherwise the finite-size effects lead to overestimation of the mutual information [45]. A more sophisticated approach is adaptive partitioning, where the size of the bins is not uniform; instead, it is chosen so that each bin contains approximately the same number of points. One such algorithm is the Fraser-Swinney algorithm [46] chosen in [28]; for a review of this and other possibilities, including kernel density estimation, see [45]. In [47] an alternative to the Fraser-Swinney algorithm was presented, which was reported to achieve comparable performance as the original method while requiring just 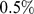 of the computational time. Further, it has the additional advantage of providing an explicit calculation of the probability of the null hypothesis that X and Y are independent.

These reasons support the choice of the aforementioned adaptive algorithm [47], which has been re-implemented and adapted in MIDER. Specifically, it has been augmented so that it calculates not only the mutual information between a pair of variables but also the joint entropies of pairs, triplets, and 4-tuples of variables, that is 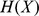, 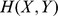, 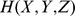, and 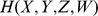, which may be required at the entropy reduction step.

#### Normalized mutual information

A characteristic of mutual information is that its range of values is in principle unknown. A number of normalizations have been proposed in the literature. An early one was the definition by Linfoot [48], with values ranging from 0 to 1:
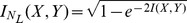
(10)


In [25] a normalization was introduced in the context of analyzing large-scale gene expression data:
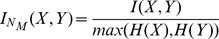
(11)


The distance measure is then defined as 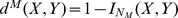. This normalization has two advantages: (1) the distance is between 0 and 1, and (2) it guarantees that 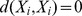.

Studholme et al [49] proposed an overlap invariant entropy measure in the context of 3D medical image alignment. It was defined as
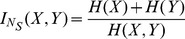
(12)which can have values between 1 and 2.

MIDER lets the user choose between any of these normalizations or the standard definition of mutual information. While normalization changes the numerical range of the distance matrix, in practice its effects on the reconstructed network are very small.

Furthermore, the user can choose between the classic definition of mutual information (Equation (3)) and the nonextensive version (Equation (8)). The default choice is the classic one; the nonextensive definition can be used if there are reasons to believe that the underlying system is more suited to it.

#### Plots of the time-lagged mutual information

**Figure 2:**
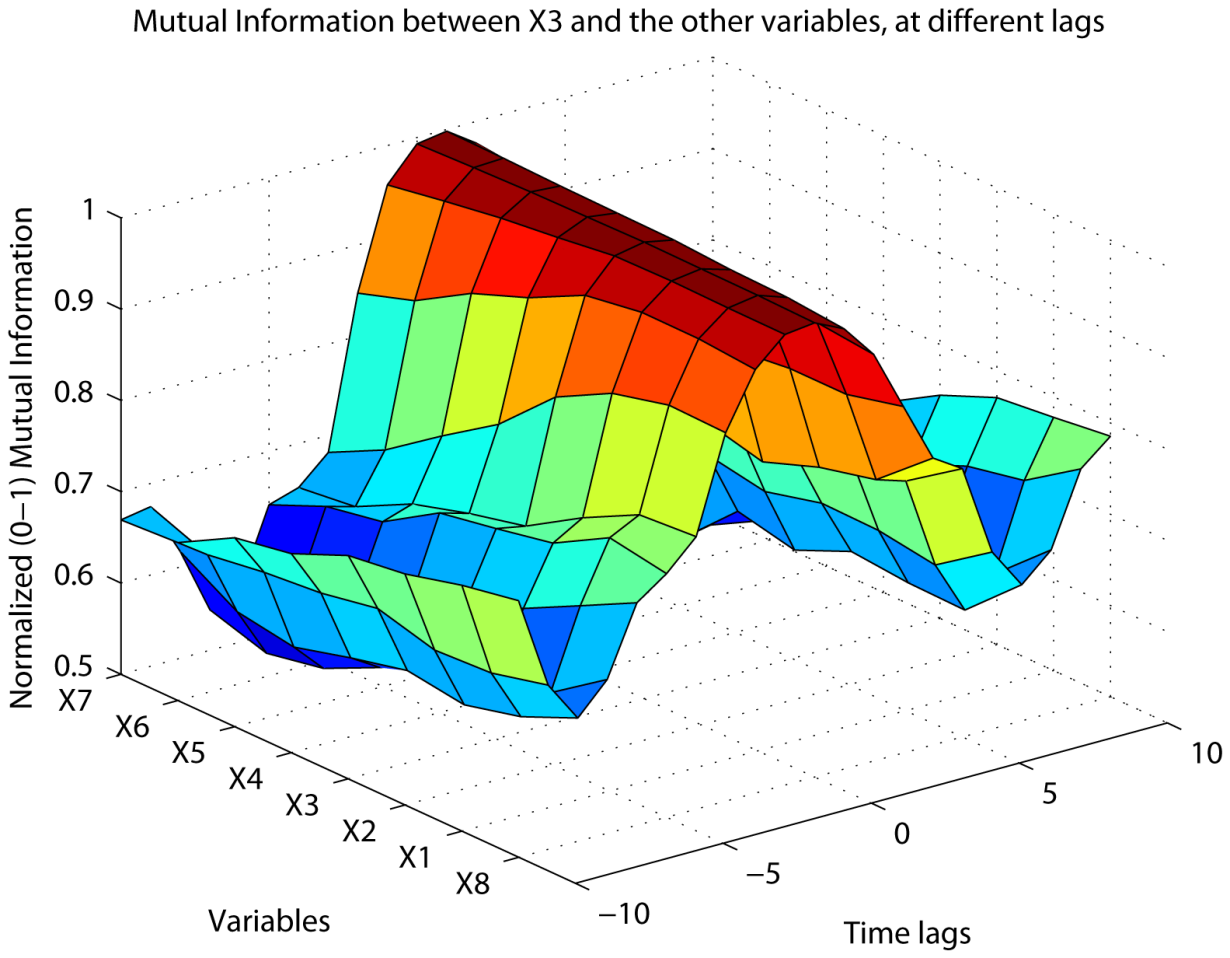
MI plot. One of the MIDER outputs, shown for Benchmark B2: a 3D plot of the mutual information between a variable (X3) and the rest, for different time lags between variables.

MIDER generates 3D plots of the mutual information between every variable and all the others, for every time lag considered. They are a graphical representation of the time-varying dependency between variables. To make visualization easier, the mutual information is normalized to the range [0,1] according to Equation (10). An example is shown in Figure 2.

### Defining the distance between variables

MIDER uses mutual information 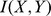 as a measure of statistical dependence to define a distance between the variables 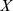 and 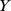. The measure of statistical closeness between two variables is the number of states jointly available to the two variables – the size of the support set – compared to the number of states available to them individually. The support set of a distribution is the smallest closed set whose complement has probability zero; it may be understood as the points or elements that are actual members of the distribution. We denote the support set of a continuous variable by 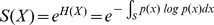. Following [28], we define the distance between two variables as the support set of the two variables divided by the product of the support sets of each variable:
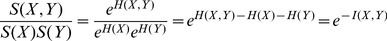
(13)


If time series data is available, the mutual information between two variables 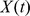 and 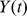 can be calculated for different delays 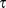, 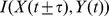. The distance used in MIDER is the minimum of Equation (13) regardless of 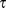:
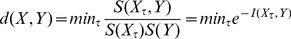
(14)which is the same distance that was defined in eq. (4). Multidimensional Scaling (MDS), a tool for representing high-dimensional data in a reduced number of coordinates, is then applied to the distance matrix for visualization purposes. MIDER uses MDS to generate a two-dimensional configuration of points representing each of the species. This 2D plot gives an indication of the likelihood of connections: the closer two species appear in the map, the more likely it is that there exists a link between them. However, in the presence of indirect relations the closeness can be misleading. To help in distinguishing direct and indirect relations, MIDER carries out entropy reduction as detailed in the next subsection.

### Detecting indirect interactions with entropy reduction

MIDER implements an entropy reduction procedure that is inspired by the one proposed in [27], [28], which was described in the Background subsection as part of the ERT method. As has been already explained, ERT seeks to determine if the variation in a variable Y can be explained by the variations in other variables in the system. The outcome of ERT would be the list of species **X** with which a given species Y reacts, in order of the reaction strength. Note that neither the EMC nor the ERT methods are publicly available. Indeed, to the best of the authors' knowledge the ERT method is a just a theoretical proposal, which has never been implemented or tested. Hence the use of the conditional tense to refer to its results. The mathematical formulation stems from the observation that, if a variable Y is completely independent of a set of variables **X**, then 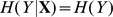; otherwise 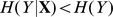. By iterating through cycles of adding a variable 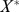 that reduces 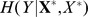, ERT would yield an ordered set of variables that control the variation in Y. Theoretically, ERT stops iterating when it stops explaining any more of Y with new X's. In practice, entropy values are estimated from data, and are therefore an approximation. Since their precision is limited, this theoretical condition is not appropriate as a termination criterion in real applications. MIDER carries out several entropy reduction rounds, and in each one follows this practical guideline to consider a connection as true and not as an artifact: for hypothesizing that a species 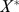 is connected with 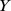 (which has already been predicted to be connected with a subset X*), its inclusion must reduce the entropy by a proportion at least equal to 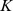. That is, a link between 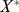 and 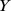 is predicted if and only if
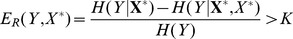
(15)where 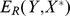 is the reduction in the entropy of 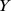 due to 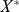, and 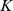 is a threshold that may be fixed by the user or (by default) calculated automatically as a function of the entropy values. By default 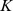 is set to a value which is obtained from the maximum reduction in 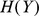 achieved by any variable 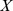, as follows:



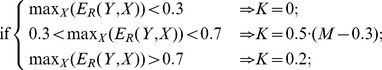



The numerical values, such as the upper limit of 0.2, were empirically chosen; this tuning was carried out with the datasets used in the Results section, for which the above rule provided good results.

Note that other measures of entropic reduction have been proposed elsewhere for similar tasks, and could also be used at this step. For example, in the area of machine learning the concept of variable relevance, defined as the relative reduction of uncertainty of one variable due to the knowledge of another, was formalized [50] in the context of feature subset selection as
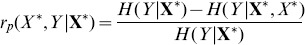
(16)


MIDER implements the entropy reduction step according to Equation (15), which was proposed in [27]. A limitation of entropy reduction is that a large amount of data is required to obtain reliable estimations of joint entropies of many variables. This was acknowledged for ERT in [27], [28], where it was noted that for multivariate Gaussian distributions the amount of data needed increases exponentially with the number of variables. Hence, when reconstructing large systems one can generally not aspire to inspect all of the possible combinations of reactants for a given species. However, this is generally not necessary, since in practice a species reacts only with a reduced number of other species. Thus it is feasible to do a limited reconstruction where the *m* most important reactants are found. MIDER is programmed to detect up to 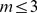 connections, which entailes estimating joint entropies of up to 4-tuples of variables 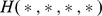 for different time lags. Since this is the most computationally expensive part of the method, it may be useful to limit the calculations to 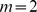 (default setting).

### Strength and causality of interactions

There are several ways of estimating the strength of an interaction between two variables 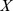 and Y. To begin with, the distance 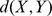 defined in Equation (14) may serve as a first indication: the smaller the distance, the stronger the interaction. To consider connections involving more than two variables, it is useful to resort to the two-dimensional map provided by MDS. For example, if three variables appear very close to each other in the map as opposed to the remaining variables in the system, this may indicate that they participate in the same reaction (for the case of chemical species). This criterion, albeit reasonable, does not take into account the possibility of indirect interactions. Hence, if three variables 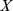, 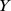, and 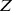 are very close, this criterion would predict links between the three variable pairs 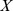
–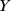, 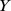
–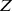, and 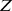
–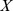. However, it may be the case that only 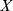
–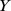 and 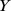
–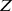 are connected, and that 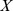 and 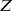 are only linked indirectly through 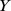. This is the motivation behind the entropy reduction step presented in the previous subsection. To help in visualizing this, MIDER gives further indications of the interaction strength by drawing links of different width between variables. The width is proportional to the entropy reduction, 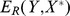. As has been already mentioned, the entropy reduction step requires large amounts of data, which can limit its accuracy in some cases. Therefore it is wise to treat its output as a complement of the distance map and not as the only criterion.

Links between variables are plotted as arrows, which represent directional (causal) relationships. Inferring causality is a subtle matter, with deep theoretical implications, and currently an open problem in biological applications [51]-[55]. Mutual information is undirected, and most information theoretic methods do not assign causality to the inferred interactions. An exception is TD-ARACNE [35], which exploits time series data to establish causality of interactions from the order in concentration changes. This idea was already present in the CMC method [29], [30], which ordered the species according to the temporal ordering of their correlation maxima. It is also possible to retrieve this information from MIDER, which, as has been already mentioned, generates plots of the mutual information (instead of correlation) between variables for different time lags. The time lags that yield the maximum mutual information between variables are reported and stored in the Output structure, in the field “Output.taumin”. MIDER assigns causality to the inferred links by calculating the transfer entropy between variables. The transfer entropy, 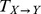, is a non-symmetric measure of causality introduced by Schreiber [56], which quantifies the reduction in the uncertainty in future values of 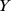 obtained by knowing the past values of 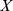, given past values of 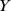. Similarly to the aforementioned entropy reduction used by MIDER, the transfer entropy is also based on time-lagged conditional entropies, and it may be expressed as a function of them as follows [57]:
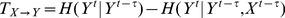
(17)


For every pair of variables (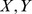) for which a link is predicted, MIDER calculates the two transfer entropies (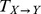, 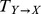), and assigns causality in the direction corresponding to the maximum of the two.

## Results and Discussion

The performance of MIDER has been evaluated with the seven benchmark problems listed in Table 1. They include examples of the three main types of cellular networks: metabolic, protein signaling, and gene regulatory networks. For two of them experimental data was available; in the remaining cases pseudo-experimental data was generated and used as the input of the reverse-engineering procedure. The results were compared to those obtained with four state of the art methods based on mutual information. We chose methods capable of detecting indirect interactions, and for which an implementation was publicly available. Based on these criteria, we selected CLR [20], ARACNE [32], [33] – which are arguably the two most widely used information theoretic methods –, and MRNET [37], all of which are implemented in the R package MINET [38]. We also tested the time-delay version of ARACNE, TD-ARACNE [35]. In those cases in which other comparisons were of interest, we also discussed other methods, namely CMC [29], [30] and MI3 [39].

**Table 1:** Benchmarks.

Number	Description	Publication	Type	Data	Data points	Variables
B1	Glycolytic pathway	[30]	Metabolic	Real	57	10
B2	8 species mechanism	[28]	Metabolic	Simulated	250	8
B3	4 species mechanism	[27]	Metabolic	Simulated	100	4
B4	IRMA benchmark	[59]	Genetic regulatory	Real	125	5
B5	MAPK cascade	[60]	Protein signaling	Simulated	210	12
B6	DREAM4 10 genes–1	[61]	Genetic regulatory	Simulated	105	10
B7	DREAM4 100 genes–1	[61]	Genetic regulatory	Simulated	210	100

List of the benchmark problems used in the comparisons.

To carry out objective comparisons between inference methods it is necessary to have quantitative measures of their performance. Two common measures are precision (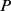) and recall (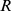), which are defined as follows. Let 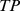 denote a true positive prediction, 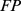 a false positive, 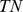 a true negative, and 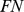 a false negative. Then precision and recall are
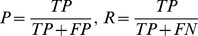
(18)


Other common measures are the true positive rate (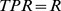) and the false positive rate (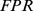),
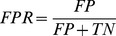
(19)


Most inference methods have some tunable parameter that represents the minimum strength that an interaction must have in order to be considered real, and not an artifact of the data. By changing this threshold and recording the different outcomes of a method, one can plot either Precision-Recall curves (PR), which show how 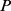 changes as a function of 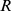, or Receiver Operator Characteristic curves (ROC), which plot 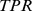 as a function of 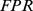. The area under precision-recall curves (AUPR) and area under ROC (AUROC) condense the information captured by the curves in a single scalar measure. It has been argued [58] that PR curves are more informative than ROC curves, which can give an excessively optimistic picture of an algorithm's performance. The reason is that a method with a seemingly good ROC curve can have a very large 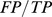 ratio, and therefore low precision. Hence in this paper we use precision, recall, and AUPR as performance measures.

Precision-Recall curves provide quantitative measures of a method's performance for a variety of settings. However, they do not give information about which performance is to be expected with the method's *default* settings, the ones that will be typically used in absence of further knowledge about the problem. Since not all methods apply the threshold in the same way, it may happen that a method with an apparently good PR curve gives a poor result (e.g. very good recall, but with low precision, or vice versa) when used with “out-of-the-box” settings. To take this into account, with the aim of avoiding unfair comparisons, we reported not only the PR curves and the AUPR value but also the (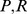) values obtained with default, out-of-the-box settings.

While MIDER and TD-ARACNE infer interaction direction, ARACNE, MRNET, and CLR do not. To enable direct comparison of these methods, we do not take direction into account when classifying a link as true or false.

Previous evaluations of network inference methods, such as the ones carried out in the DREAM initiative, have stressed the importance of the “wisdom of crowds” [14], [16]. While no single method was found to be optimal for every problem, the integration of the outcomes of all methods in a “community prediction” provided a consistent performance across all datasets. This observation prompted us to investigate whether this would also be the case for the set of problems and methods compared here. With this aim we created a community prediction for every benchmark by averaging the connection strengths yielded by each method and applying a threshold (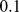) to the result.

**Figure 3:**
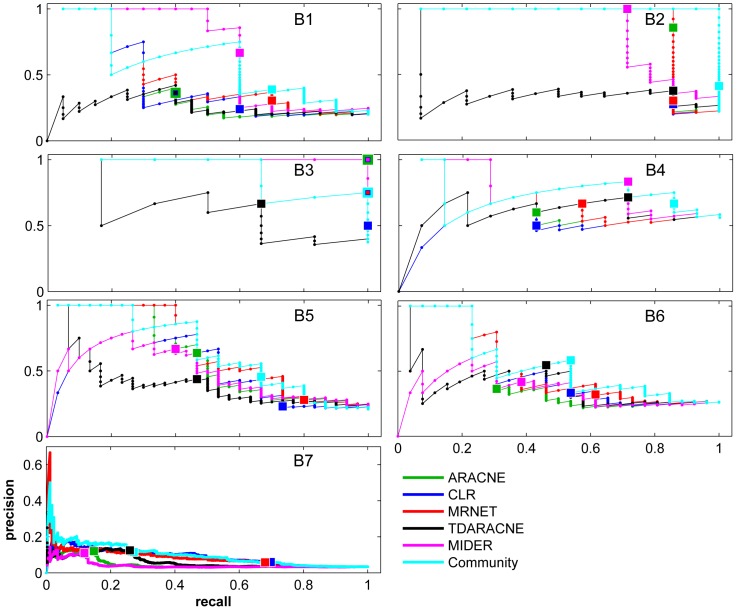
Precision-Recall curves. PR curves (recall in horizontal axis, precision in vertical axis) of all the benchmarks (B1–B7) for five network inference methods (ARACNE, CLR, MRNET, TDARACNE, and MIDER) and for the community prediction. Solid lines and small dots correspond to the (P,R) values obtained by changing the threshold for detecting interactions. Large square points correspond to the (P,R) values obtained with the default (out of the box) settings of each method.

**Figure 4:**
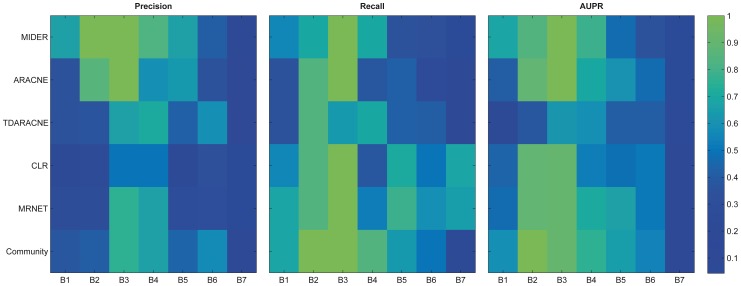
P, R, and AUPR. The color maps show precision (left panel) and recall (central panel) achieved by each method and for each benchmark with its default settings, as well as the area under precision-recall curve (AUPR, right panel). Numerical values are in the range [0–1], and are represented in colors according to the scale in the right (green  =  good, blue  =  bad).

Figure 3 shows Precision-Recall curves of the five algorithms (and the community prediction) for the seven benchmark problems, including default (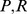) values. The same information is provided using two-dimensional color maps in Figure 4. In accordance with previous comparisons reported in the literature, no algorithm was the best performer for all problems. MIDER was the best performer –best precision and recall– for benchmarks B3 and B4, and provided the result with highest precision for B1, B2, and B5. For the genetic networks of B6 and B7 it did not provide the best precision nor the best recall, but ranked in intermediate positions among other methods. On average, we found the performance of MIDER to be at least comparable to that of the other methods.

It should be noted that the community prediction turned out to be comparable to the best result obtained by any method in six of the seven benchmarks. In other words, the community prediction is in the Pareto front of non-dominated solutions for those cases: no method has simultaneously better precision and better recall than the community. Thus, the community prediction is the optimal trade-off between precision and recall for a given weight of 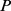 and 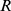 (although not for every possible weight). The only exception is benchmark B3, for which, exceptionally, two methods (MIDER and ARACNE) provided perfect reconstructions, so the addition of less accurate results made the community a worse solution than the one provided by those methods.

These results show, on the one hand, that MIDER is a good option for network inference in a variety of settings, and on the other hand, that it is advantageous to take into account the outcomes of several algorithms. The following subsections describe the benchmark problems and analyze the results in more detail.

### Benchmark B1: glycolytic pathway

**Figure 5:**
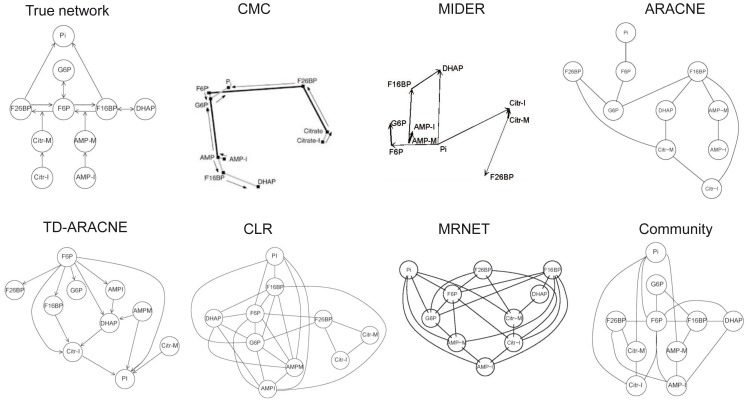
Benchmark B1. First reaction steps of glycolysis.

As a first example we considered the first steps of the glycolytic pathway, which are depicted in the upper left panel of Figure 5. The problem of reverse-engineering this system – a chemical reaction network of realistic size – was chosen in [30] as a way of demonstrating the feasibility of the CMC method. With that aim, an experiment was carried out in a continuous-flow, stirred-tank reactor (CSTR). Experimental time-series data was obtained for the concentrations of ten species: Pi, G6P, F6P, F16BP, F26BP, and DHAP, as well as the input and reactor concentrations of citrate and AMP. The sampling period was 13 minutes, and the overall number of sampling instants was 57. The data is publicly available at http://genomics.lbl.gov/?page_id=44 as part of the Deduce software package. We remark that, although the MIDER method is theoretically capable of detecting more complicated relationships between variables than CMC, it also requires more data points to carry out this task reliably. Thus, it is useful to demonstrate that it produced similar results to CMC (shown in Figure 5) in cases such as this one, when the available data was limited (in the next example we show a situation where the MIDER method improved the CMC prediction for a system for which more data was available). Among the benchmarked methods, MIDER (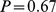, 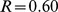) yielded the highest precision with out-of-the-box settings, outperforming ARACNE, TD-ARACNE (both of which achieved 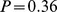, 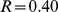), and CLR (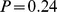, 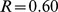). MRNET (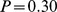, 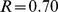) yielded the highest recall, although with low precision.

### Benchmark B2: enzyme-catalyzed reaction pathway

**Figure 6:**
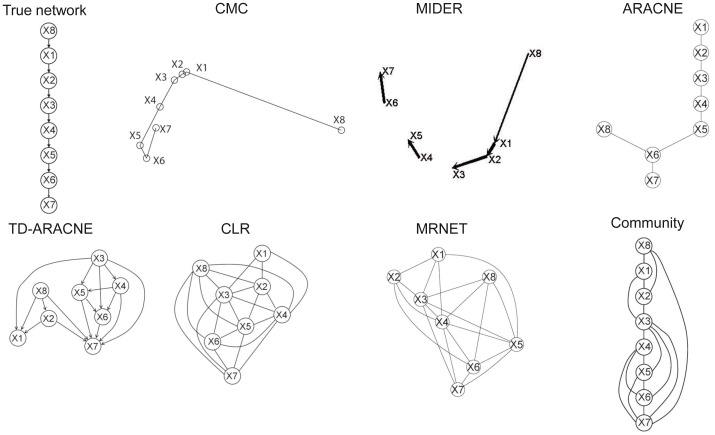
Benchmark B2. Reaction chain with 8 species.

As a second example we chose a simulated metabolic pathway, the chemical reaction network represented by
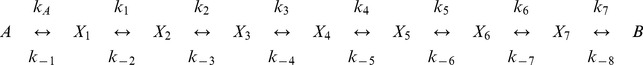
(20)where species A and B are kept at constant concentrations, 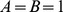. The step 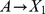 is enzyme catalyzed with a rate coefficient 
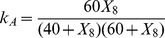
, where 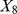 is the input species; its concentration is varied randomly. The remaining steps are first order reactions, with forward rates 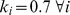 and backward rates 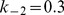, 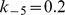, and 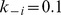 otherwise. This example was introduced in [28] to illustrate the difficulties that arise from the self-inhibition of the enzyme catalysis by 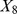. The quadratic dependence of 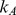 on 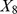 creates a strong nonlinearity that complicates the reverse engineering of this model with a correlation-based method such as CMC, which was designed to quantify linear interdependence. However, CMC still recovered correctly the mechanism (see Figure 6), although it predicted a very weak link between 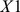 and 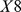, and showed a wrap around in the 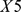
–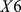
–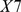 part of the chain. The automatic reconstruction yielded by MIDER, in contrast, did not present those issues, although it did not predict the 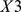
–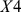 and 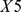
–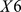 links directly, since their interaction strength was slightly lower than the default threshold. However, these links can be clearly inferred by visual inspection from the 2D entropic distance map. Without adding these links, MIDER yielded a perfect precision (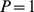) and a recall of 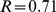. ARACNE yielded higher recall (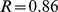) but lower precision (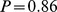), due to the false prediction of a link between 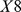
–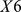 instead of 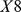
–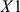. TD-ARACNE, CLR, and MRNET yielded several false positives, leading to a good recall (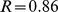) but also to low precisions (in the range 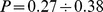). It should be noted that this benchmark uses artificial data. We generated data corresponding to 250 time points, instead of the 2000 used in [28], a restriction that makes this problem more realistic and more complicated than the one originally published.

### Benchmark B3: small reaction pathway

**Figure 7:**
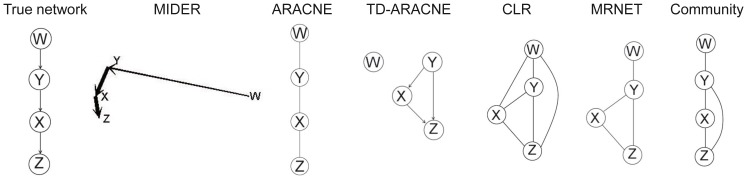
Benchmark B3. Reaction chain with 4 species.

Next we considered the following small linear chain of reactions,
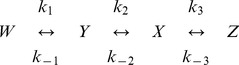
(21)where reaction 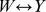 is much weaker than the rest: 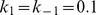, while 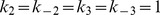. The use of this system was proposed in [27], [28] as a target application for the Entropy Reduction Technique (ERT). The difficulty posed by this system is caused by the different values in the kinetic constants. Due to them, both correlational and entropic distances between variables are small for the 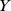
–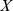, 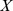
–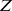, and 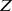
–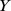 pairs, while the 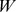
–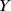 distance is large. The resulting configuration of points obtained with MIDER is shown in Figure 7. Note that, if the method took into account only the distances between points, it would predict links between 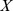
–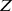, 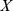
–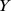, and (incorrectly) 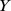
–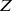; since the distance between 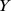 and 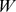 is large, only a weak link between them – or no link at all – would be predicted. The ERT method was proposed in [27], [28] to improve the predictions in this situation: it was hypothesized that by calculating conditional entropies ERT would establish that, though 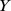 is strongly dependent on 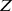, all of the dependence (or, due to lack of precision, most of it) is due to 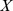. Despite being proposed, however, ERT was never tested. The implementation of an entropy reduction procedure included in MIDER confirmed the aforementioned hypothesis: not only did it predict a link between 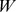 and 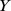 and another one between 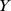 and 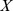, it also estimated that the link between 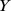 and 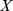 was stronger than the one between 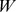 and 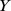. If the variables 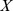, 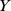, and 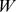 are chemical species, as in this case, this may be taken as an indication that the kinetics between 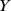 and 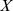 are faster than between 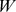 and 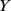. For this benchmark both ARACNE and MIDER achieved perfect precision and recall (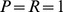). It must be noted that the data was generated by changing the concentration of 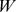 randomly; thus 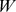 acted as an input species whose variation was propagated to 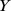, then to 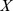, and finally to 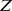. Therefore, although the reactions are reversible (and hence the 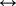 symbol in Equation 21) there is a directionality in the interactions that should be ideally inferred by the methods. MIDER predicted correctly the direction of the 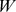
–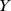 and 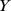
–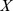 links, and incorrectly predicted a bidirectional interaction between 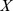
–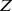. ARACNE, however, does not infer directionality of the interactions. CLR, TD-ARACNE, and MRNET provided incorrect reconstructions. Some network inference methods make other assumptions about the connectivity of the network, often based on considerations about the architectures that are common in gene regulatory networks. This choice may limit their generality. For example, the MI3 method mentioned in the Introduction uses a metric (Equation (6)) designed for detecting cooperative activity between regulators. It assumes that every species (target) is linked with two regulators, which causes false positives in cases such as this.

### Benchmark B4: IRMA

**Figure 8:**
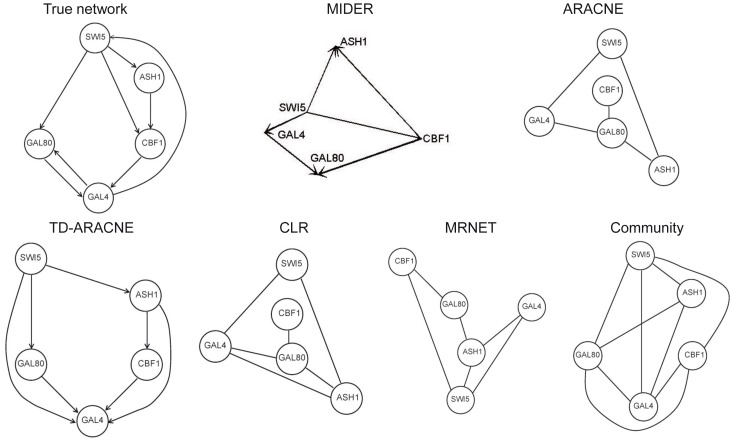
Benchmark B4. IRMA.

IRMA (In vivo Reverse-engineering and Modeling Assessment) [59] is a yeast synthetic network for benchmarking reverse-engineering approaches. It consists of five genes that regulate each other through several interactions. It is particularly interesting as a benchmark because it is an engineered system, which means that the true network is known, and at the same time the system outputs can be measured in vivo, instead of just simulated in silico. A dataset consisting of time series and steady-state expression data after multiple perturbations is available; for the network inference purposes the time-series data was used. Figure 8 shows the results of the different methods. The outcome of TD-ARACNE had already been reported in the original publication [35], since IRMA was one of the benchmark problems selected to demonstrate the performance of that method; we repeated the calculations and obtained the same result (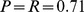). MIDER achieved the same recall as TD-ARACNE with slightly higher precision (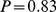). According to the precision-recall metrics the worst result was the one obtained by CLR (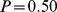, 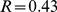); ARACNE and MRNET outperformed CLR but fared worse than MIDER and TD-ARACNE.

It must be noted that the five methods predict a link between SWI5 and GAL4, which does not exist in reality (SWI5 is linked to GAL4 only indirectly, through GAL80). GAL4 and GAL80 form a complex, and it was already acknowledged in the original publication [59] that these two proteins may indeed be considered as a single component for reverse engineering purposes: since no protein data is available, network inference is carried out with mRNA concentration data, and it is unlikely that the real protein–protein interactions are correctly recovered.

### Benchmark B5: MAPK cascade

**Figure 9:**
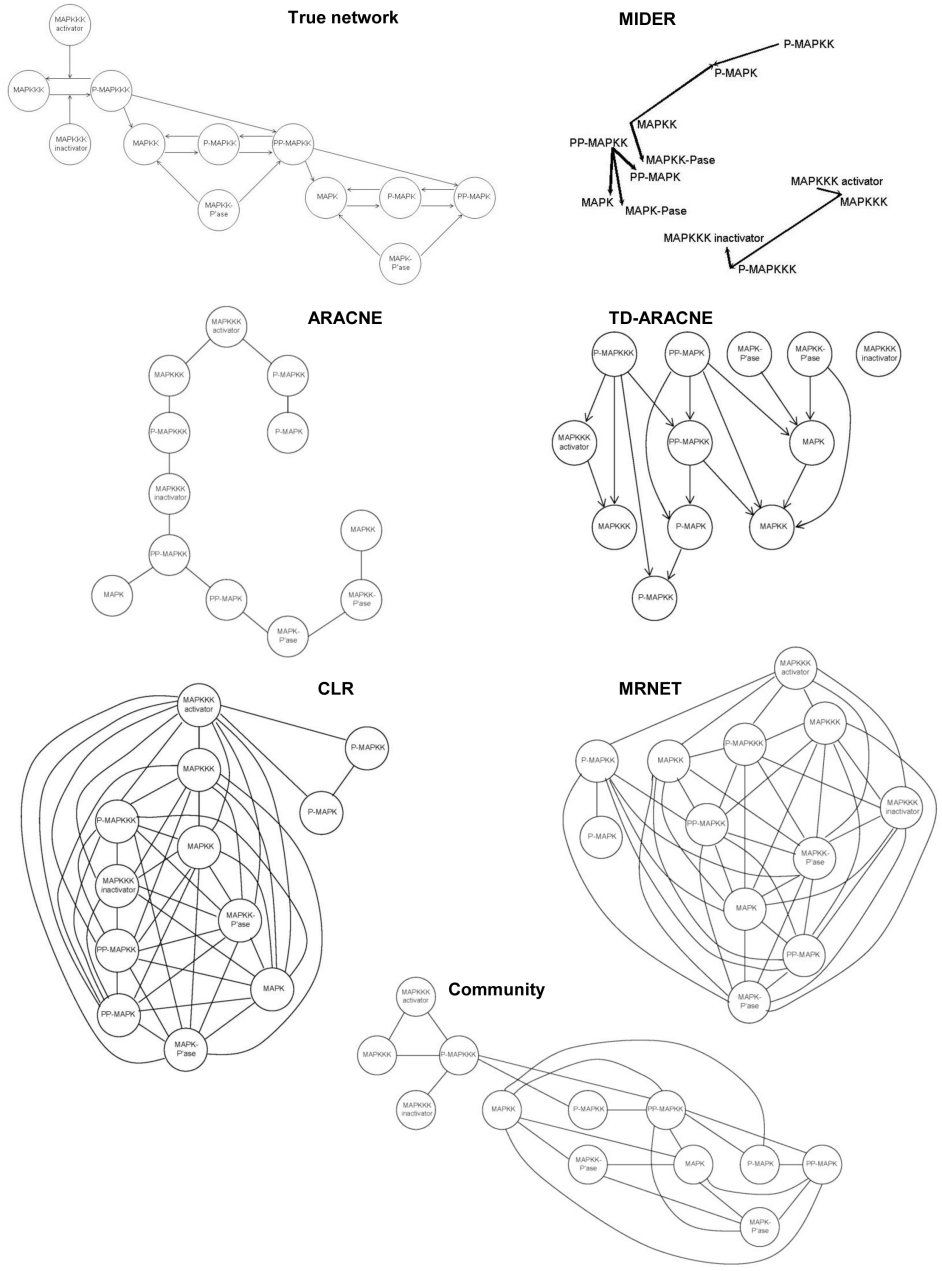
Benchmark B5. MAPK cascade.

The classic Mitogen-Activated Protein Kinase model presented by Huang & Ferrell [60] is a highly conserved series of three protein kinases implicated in diverse biological processes. It exhibits a highly nonlinear (“ultrasensitive”) behavior [60], converting graded inputs into switch-like outputs. The cascade as a whole behaves like a highly cooperative enzyme, even though none of the enzymes in the cascade are regulated cooperatively. This benchmark was more difficult to recover than the previous ones, due to a larger number of network nodes and more complex interactions. The reconstructions, shown in Figure 9, differ largely from one method to another, with clear trade-offs between precision and recall: MIDER yielded the highest precision (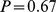) but with the lowest recall (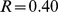); MRNET, on the other hand, yielded a high recall (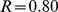) with low precision (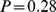), and so did CLR (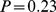, 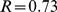). The P-R metrics of the results provided by ARACNE and TDARACNE were intermediate between those of MIDER and MRNET. Although none of the benchmarked methods generated a really good approximation of the complex network, all of them succeeded in predicting the linking between the MAPKKK activator, MAPKKK, and P-MAPKKK (and, with the exception of TD-ARACNE, also with the MAPKKK inactivator); that is, the most upstream part of the network. Reconstructions of the rest of the network, however, are much less accurate. Interestingly, the three levels of the cascade can be distinguished in the spatial configuration yielded by MIDER, which consisted of three distinct groups of species (although it confused P-MAPK and PP-MAPKK).

### Benchmarks B6 and B7: DREAM4 in silico gene networks

Finally, we tested the methods using benchmark problems generated for the DREAM4 in silico network challenge (http://wiki.c2b2.columbia.edu/dream/index.php/D4c2). This network inference challenge consisted of different subchallenges, which aimed at reverse engineering genetic networks of sizes 10 and 100. The artificial networks were generated as reported in [61], [62]. We picked one network of each size: network 1 from the DREAM4_InSilico_Size10 dataset, and network 1 from the DREAM4_InSilico_Size100 dataset. Since these are artificial networks their representations have no biological meaning, and hence are not pictured here.

The performance of all the five methods compared in this section was relatively modest for the network of size 10. Precision values ranged from 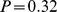 (MRNET) to 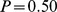 (TD-ARACNE), and recall from 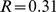 (ARACNE) to 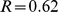 (MRNET). The reconstruction obtained with MIDER yielded intermediate values (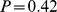, 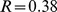).

For the network of size 100 all methods obtained poor results. We found a clear distinction between methods that focused on precision (ARACNE, TD-ARACNE, MIDER) and methods that focused on recall (CLR,MRNET). Methods from the first group achieved precisions in the range 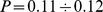 and recalls in the range 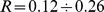, while methods in the second group yielded even lower precision values (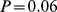), but with recalls in the range of 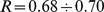.

## Conclusions

The present work has introduced a methodology for network inference called MIDER. It is based on information theoretic concepts, and combines the use of mutual information-based distances and entropy reduction. It outputs a visual representation of the inferred system, as well as estimates of the strength and directionality of the interactions, and time-lagged plots of the mutual information between variables. Among other options, it offers the possibility of choosing from different normalizations of the mutual information, and even a nonextensive version.

One of the strengths of MIDER is its generality: it makes no assumptions about the characteristics of the network, which makes it suitable for inferring connections in systems where little is known. Indeed, the only necessary input is the experimental data. Another advantage of the method is that, although it has some tunable parameters that can be modified if desired, it requires no expertise from the user. Due to the adaptive nature of its subroutines, its default settings provide good results for a variety of problems. It has been tested on seven different benchmarks including metabolic, gene regulatory, and protein signaling networks, and has performed well when compared to other state of the art techniques.

Regarding its theoretical foundations, a strength of MIDER is its ability to detect multiple interactions and avoiding false positives. It ranked first in precision among the tested methods in five of the seven benchmark problems considered, and achieved precision scores close to the best performer in the other two. Since in every reverse engineering method there is a trade-off between precision and recall, this emphasis in precision entails that MIDER can yield low recall for large-scale problems. However, for smaller-scale networks (up to ten nodes in our tests) it manages to obtain simultaneously high precision and high recall.

The main hurdle to surmount in order to accurately discard false positives is the need of large amounts of data, which are required if it is desired to carry out more than three entropy reduction rounds. This limitation is due to the difficulty in estimating reliably joint entropies of high dimensions (i.e., of four or more species), and is hence shared by all information-theoretic methods. For networks with a large number of components, performing more than three entropy reduction rounds may also involve high computational costs, particularly if many possible time lags are taken into account. To alleviate this burden, MIDER estimates the mutual information using an algorithm that is much faster than the one used by some of the precedent methods. Furthermore, since the related calculations are carried out in arrays and are amenable for parallelization, this limitation can be easily overcome. As a future development we plan to implement a parallel version of MIDER.

We hope that MIDER will be a valuable addition to the existing methodologies for network inference, either by itself or in combination with other algorithms to create a community prediction. To facilitate its use, we provide the source code along with the datasets required to reproduce the results reported in this paper. We envision that it will be particularly useful for the community of Matlab users; to the best of the authors' knowledge, this is the first time that a Matlab implementation of a comparable method is made available.

## Supporting Information

File S1 **MIDER toolbox.** This compressed file contains the MIDER toolbox, which is implemented in Matlab. It includes all the datasets used in this article, the source code of the MIDER functions, and a user manual.
(RAR)
